# The Enduring Controversy of Cervicogenic Vertigo, and Its Place among Positional Vertigo Syndromes

**DOI:** 10.3390/audiolres11040045

**Published:** 2021-09-26

**Authors:** Marcello Cherchi, Frank E. DiLiberto, Darío A. Yacovino, Sunit Das

**Affiliations:** 1 Department of Neurology, Northwestern University Feinberg School of Medicine, Chicago, IL 60611, USA; 2 Chicago Dizziness and Hearing, Chicago, IL 60611, USA; 3 Department of Physical Therapy, Rosalind Franklin University of Medicine and Science, North Chicago, IL 60064, USA; frank.diliberto@rosalindfranklin.edu; 4 Department of Neurology, Dr. César Milstein Hospital, Buenos Aires C1221 ABE, Argentina; dyacovino@gmail.com; 5 Memory and Balance Clinic, Buenos Aires C1424 AFC, Argentina; 6 Division of Neurosurgery, St. Michael’s Hospital, University of Toronto, Toronto, ON M5B 1W8, Canada; sunit.das@utoronto.ca; 7 Keenan Research Centre for Biomedical Science, Toronto, ON M5B 1W8, Canada; 8 Arthur and Sonia Labatt Brain Tumor Research Centre, Hospital for Sick Children, Toronto, ON M5G 1X8, Canada

**Keywords:** vertigo, cervicalgia, proprioception, multisensory integration, migraine, vestibular testing, oculomotor testing, vascular imaging, physical therapy

## Abstract

The idea of cervicogenic vertigo (CV) was proposed nearly a century ago, yet despite considerable scrutiny and research, little progress has been made in clarifying the underlying mechanism of the disease, developing a confirmatory diagnostic test, or devising an appropriately targeted treatment. Given the history of this idea, we offer a review geared towards understanding why so many attempts at clarifying it have failed, with specific comments regarding how CV fits into the broader landscape of positional vertigo syndromes, what a successful diagnostic test might require, and some practical advice on how to approach this in the absence of a diagnostic test.

## 1. Introduction

Quotidian medical practice does not usually contemplate a diagnosis from the perspective of ontology (is this really there?), epistemology (how do we even know that this is really there?), and nosology (what is actually diseased?), but cervicogenic vertigo (CV) probably warrants such considerations.

Investigations of CV often appear motivated by the frequently encountered clinical scenario of a patient with neck symptoms and dizziness, in whom no other cause for the dizziness has been identified. Since mere co-occurrence does not prove causality, a skeptical audience would understandably eschew regarding CV as a “diagnosis,” and prefer the more neutral term, “syndrome”.

Discussions of cervicogenic vertigo (CV) usually characterize the idea as “controversial” [1,2,3], and acknowledge that the lack of a diagnostic test contributes to the controversy [3,4,5,6].

Most medical phenomena that eventually come to be accepted as diagnoses began as unproven ideas; as hypotheses that required testing. Since the absence of proof is not proof of absence, we should remain receptive to the possibility of progress on this topic, and that the controversy surrounding CV may ultimately be resolved.

With these points in mind, we shall review *why* CV is controversial, beginning with an appraisal of candidate mechanisms for its pathophysiology, how these mechanisms could be tested, why tests have failed, and a more general discussion of why it has proven so difficult to devise a sensitive and specific test. We conclude with a brief review of treatments.

The literature regarding CV spans nearly a century, and in reviewing this, we have been struck by the degree to which paraphrasing the text of a previous author appears often to result in changing the earlier article’s intent. In the interest of accurately conveying the thought of previous authors, we have elected generous use of direct quotations in the present review.

## 2. Assumptions, Definitions, and Conditions

For purposes of this discussion, we shall make the initial simplifying assumption that cervicogenic vertigo (to be defined presently) is the only source of symptoms—though later we shall also mention why this assumption is faulty.

We take the term “vertigo” in its technical sense as referring to a kinetic illusion; a discrepancy between perceived versus actual motion/stasis. While we would prefer a more neutral term such as “disequilibrium” or “dizziness,” the word “vertigo” has become quite entrenched in the literature since Ryan and Cope’s 1955 paper on the subject [7]. Some readers may take the term “vertigo” to refer more narrowly to a sensation of rotation, but many authors note that such a sensation is actually uncommon in CV [6,8]. The term “cervicogenic” designates that the underlying mechanism of vertigo arises from (is “generated” by) a problem in the cervical region—in other words, the definition requires a causal relationship, whereby a cervical problem provokes vertigo.

Most research on CV requires that (explicitly or implicitly) in order to entertain a diagnosis of CV, either (1) neck symptoms must be present (pain; head-on-neck and/or neck-on-trunk movement that is limited, excessive, uncontrolled, unintended, irregular, etc.), and these neck symptoms must temporally overlap with the symptom of vertigo; or (2) there is a history of neck injury that precedes the development of the symptom of vertigo; or (3) both [2,8,9].

## 3. Immediate Problems

When one attempts to apply these assumptions, definitions, and conditions to clinical cases, several problems become immediately apparent.

The first problem pertains to the relationship between cervical disease and vertigo; while the definition of CV requires that cervical disease be the cause of vertigo, there are, of course, other possible relationships.

One possibility is that the temporal overlap of symptoms may hold, but the causal relationship does not—in other words, the relationship is one of coincidence rather than causality [10]. This possibility merits consideration because neck pain and vertigo are each very common human experiences, and even when each symptom results from an independently occurring etiology, the likelihood of temporal overlap (coincidence) by random distribution is not small. Neck pain is common and appears to be increasing; the prevalence among adults aged 25–84 in the US was 14.8% in 2002 and 17.2% in 2018 [11]; similar demographics are reported in other countries [12]. In the US, dizziness and vertigo accounted for 20.6 million ambulatory care visits per year in 2013–2015 [13], and for approximately 4 million emergency department visits in 2011 [14]. Thompson-Harvey and Hain capture this idea by noting that “The main clinical problem in diagnosing cervical vertigo is that symptoms of subjects who have both neck disorders and dizziness may overlap… In other words it is difficult to differentiate between the chance coincidence of arthritis of the neck and dizziness, from the situation where arthritis of the neck causes dizziness” [9].

Another possibility is that the temporal overlap of symptoms may hold, but the relationship is reversed; instead of neck pathology causing vertigo, the vertigo causes the neck symptoms. Patients with vertigo of any cause often unconsciously make compensatory postural adjustments, and neck symptoms may ensue [6,15]; in other words, the neck symptoms may be an effect of the vertigo, rather than its cause.

The second problem pertains to the relationship between CV and neck injury. Hain states that, “inner ear disorders are rare after neck trauma” [5], citing Mallinson and Longridge [16]. However, most injuries are not so focal as to affect the neck in isolation; in fact, whiplash—the most common neck injury, in whose context cervicogenic vertigo is suspected—is usually not a “pure neck injury.” Yacovino and Hain note that “Postwhiplash vertigo can combine several mechanisms. At the ear level, the otolith system is prone to suffer inertial damage” [6], and several authors have noted that benign paroxysmal positional vertigo can result from acceleration-deceleration injuries [12,17,18]. More broadly, “Dizziness following neck injury may be due to vestibular system pathologies, brain injury, or cervicogenic dizziness” [8], including “the ear (labyrinth contusion), the brainstem, the cortical and subcortical structures, and the vertebral arteries (traumatic artery dissection)” [6]. The analytically desirable simplifying assumption we mentioned earlier (CV is the only source of symptoms) often does not hold in the real-world laboratory of clinical medicine; neck injuries are a good example where this assumption often fails. Finally, a significant proportion of neck-injury-related CV cases is composed of whiplash injuries, and patients in many such cases are in (or are contemplating) litigation. The medico-legal dimension of whiplash injuries [19] introduces considerations beyond anatomy and physiology whose influence on symptoms (which are subjective reports) is difficult to assess, and which have the potential to complicate analysis by introducing psychological components [20], and the possibility of secondary gain.

## 4. Underlying Physiology

Discussions of CV usually assume a physiologic framework in which there is integration of multiple sensory inputs with planned output, and then there are multimodal outputs. An example of this view is evident in the following: “The results of several studies suggest that the control of posture, perception of the orientation of the body, and the location of objects in extrapersonal space requires an integration of proprioceptive, visual, and vestibular signals as well as internally generated signals related to intended head and body movements” [21], sometimes referred to as “efference copies” or “corollary discharges.” Efference copies refer to cortical constructs that serve to anticipate head-body position under a range of conditions and predict the appropriate motor response. These constructs are modified throughout life based on experienced sensory feedback.

For an individual to perceive correctly her orientation in, and movement through, space, the brain must, at some level, solve the problem of a **coordinate transformation** (mapping from the coordinate system of one frame of reference to that of another frame of reference) [22,23]. Brandt described this well:

*“It is necessary for the sensorimotor control system to know the attitude of the head relative to the body, since the vestibular system signals only head motion relative to space and head position relative to the gravity vector. The head-mounted sensory systems must transform the rotations and accelerations they sense and correctly relate their direction to the motion and attitude of the body and the center of gravity. Neck afferents provide information about head position, and make an important contribution to the control of body and sensory spatial orientation. The perception of head or trunk rotations in space would be erroneous if only vestibular stimulation or only neck stimulation was involved. However, if the two stimuli are combined (head rotations relative to the trunk), the perception of both trunk and head rotation in space reflects the true position”* [1].

Although this is a “computationally difficult adjustment between the two coordinate systems of the head and body” [6], there is nevertheless reasonable evidence that such a coordinate transformation does take place. Specifically, studies from primates “conclude that sensory vestibular signals are transformed from head-in-space coordinates to trunk-in-space coordinates on many secondary vestibular neurons in the vestibular nuclei by the addition of inputs related to head rotation on the trunk. This coordinate transformation is presumably important for controlling postural reflexes and constructing a central percept of body orientation and movement in space” [21].

A failure of **multisensory integration** (the process of reweighting and combining multiple input streams to produce a coherent perception) is the most commonly postulated mechanism underlying the idea of CV, with the failure being attributed to presumed erroneous proprioceptive cervical signals. Again, Brandt states:

*“Somatosensory signals from musculotendinous receptors in the neck and joints provide an accurate kinesthetic feedback of the extent of head and limb movements. These signals contribute to the perception of self-motion during active locomotion by converging with vestibular and visual input on multimodal neurons in the vestibular nuclei and thalamus, which project to cortical multisensory areas in the parietal lobe”* [1].

We will now review these, and other potential etiologies of disequilibrium related from the neck.

## 5. Candidate Pathophysiological Mechanisms

A number of pathophysiological mechanisms have been proposed to underlie CV. Some of the main ones are summarized in Table 1. We will discuss each in the following sections.

### 5.1. Hypoperfusion

Hypoperfusion, secondary to vascular compromise, is often discussed as a mechanism of CV. Rotational vertebral artery syndrome (RVAS), also called bowhunter syndrome, is a condition in which one of the vertebral arteries is transiently extrinsically compressed during neck rotation [24,25,26,27,28,29,30,31,32,33,34,35,36]. This appears more likely to provoke symptoms if the contralateral vertebral artery is already narrowed, such as by atherosclerotic disease. In some cases, this compression, perhaps combined with torquing of the artery, results in damage to the artery itself, such as a dissection [37]. There are a few reports of RVAS manifesting with predominantly downbeat nystagmus [38,39], though such a pattern of nystagmus is not specific for the condition. If the vascular compromise is prolonged, it may culminate in infarction [40]. The discussions of RVAS are based primarily on case reports or small case series. Despite the apparent enthusiasm in the literature, this condition probably comprises only a small proportion of cases of CV. There is no consensus on the range of effects that neck turning can have on the vertebral arteries [41], and while dynamic vascular imaging can provide corroborative evidence of RVAS, Hain [5] pointed out that “Vertebral artery blood flow is compromised with full contralateral rotation in healthy individuals,” citing Mitchell [28]; thus, vascular imaging is not specific for this condition. Further, cadaveric animal model evidence suggests typical physiologic motions at the neck produce vertebral artery strains substantially lower than the failure point (dissection) [42]. An even less common mechanism of vascular compromise occurs in Chiari malformations, in which neck rotation torques the structures at an already crowded foramen magnum [43].

Hypoperfusion secondary to an autonomic abnormality triggered by neck rotation has been discussed in several forms. Barré–Lieou syndrome, proposed by Jean-Alexander Barré [44] and Young-Choen Lieou [45], was thought to be the result of mechanical stimulation of paravertebral sympathetic ganglia during neck rotation. This theory was accepted by some early investigators [46], but ultimately “No sympathetic or vascular changes were subsequently identified that could account for these symptoms and this theory lost favor” [8], so the idea was dismissed by subsequent researchers, who state that “Positional nystagmus cannot be attributed to a disturbance of the cervical sympathetic chain as suggested by Barré” [1]; see also Foster et al. [47]. A more plausible mechanism of autonomically-mediated hypoperfusion is “head turning-induced hypotension” [48] triggered by stimulation of overly sensitive carotid sinus baroreceptors during neck rotation. This should be detectable on physical examination.

### 5.2. Anatomic

Distortion of the anatomy at the craniocervical junction, such as in patients with craniocervical instability in whom neck movements may provoke brainstem compression [43], has been postulated as a mechanism for CV. This should be detectable on imaging. Given the neuroanatomical territory involved, brainstem compression should manifest with symptoms beyond simply vertigo.

### 5.3. Oculomotor Abnormalities

Reports have documented a variety of oculomotor abnormalities occurring in association with neck rotation or neck pain; usually these appear to be abnormalities in the cervico-ocular reflex [49,50,51], but reports also describe other “Deficits in oculomotor control, such as decreased smooth pursuit velocity gain, altered velocity and latency of saccadic eye movements” [3]. Some investigators have gone so far as to say that “The smooth pursuit neck torsion test developed by Tjell et al. [52] is considered to be specific for detecting eye movement disturbances due to altered cervical afferent input” [3], but we will discuss below that this was not borne out.

### 5.4. Proprioception

Perhaps the most popular theory about CV pertains to cervical proprioception. Note that “Proprioception is not a function of the superficial neck muscles but of the deep short intervertebral neck muscles, which are extensively supplied with muscle spindles” [1], and in fact, “Of all the muscles in the body, it is the deep neck muscles that have the highest concentration of muscle spindles” [12]. As a result, “The proprioceptive system of the cervical spine… is extremely well developed, as reflected by an abundance of mechanoreceptors, especially from the gamma-muscle spindles in the deep segmental upper cervical muscles” [47], and “The dense network of mechanoreceptors in the soft tissues in this region… gives the CNS information about the orientation of the head with respect to the rest of the body via direct neurophysiological connections to the vestibular and visual systems” [3]. Specifically, “Strong connections have been demonstrated between the cervical dorsal roots and the vestibular nuclei with the neck receptors (such as proprioceptors and joint receptors) playing a role in eye-hand coordination, perception of balance, and postural adjustments” [8]. Brandt and Bronstein [15] point out that studies of various kinds of neck stimulation can alter perception. Specifically, “Unilateral electrical stimulation of the neck [53] causes deviation of the subjective vertical” and “Vibration of neck muscles, which stimulates the primary endings of the muscle spindles as if the muscle were being stretched [54] elicits an illusion of head tilt and apparent movement of a visual target [55]”.

Such data, as well as evidence from experiments involving injection of anesthetics into the neck, “leads to the current theory that cervicogenic dizziness results from abnormal input into the vestibular nuclei from the proprioceptors of the upper cervical region” [8]. While there may be compelling evidence of erroneous cervical proprioception, this in itself does not necessarily explain why that altered sensory input would manifest as vertigo. There are two plausible mechanisms.

The first manner in which erroneous cervical proprioception could manifest with vertigo is through “sensory mismatch”—which is to say a discrepancy between the erroneous input from cervical proprioception, and the correct input from vision and the inner ear [3,5,9,47].

The second manner in which erroneous cervical proprioception could manifest with vertigo is through a mismatch between intended movement (“efference copy” or “corollary discharge”) and erroneously perceived actual movement; as Brandt comments, “a multisensory mismatch would be expected to result in CV… the resultant mismatch would be maximal during active head movements (when expected and actual reafferent input do not match)” [1]. Evaluation of this possibility has been attempted with “cervical repositioning tests” [3], and “joint position error” tests [56,57].

### 5.5. Motor Mechanism

While most discussions of the mechanism of CV focus on a problem with input, the possibility of altered motor output is rarely mentioned. This hypothesis maintains that CV is due to impaired motor activity, perhaps due to incorrect modulation of motor pathways arising from an abnormality in the neck, and that this motoric impairment is truly manifesting with unsteadiness that the patient is correctly perceiving. These patients generally have normal motor examinations, so if this hypothesis is correct, then the findings may be more subtle than what is discernible on physical examination. Our group tried to evaluate this idea with triceps vestibular evoked myogenic potentials [58,59,60], on the hypothesis that vestibulospinal reflexes may be impaired, but we failed to identify any findings specific to patients whose histories were compatible with CV.

### 5.6. Migrainous Mechanism

Yacovino and Hain proposed migraine as a mechanism for CV in the form of “migraine-associated cervicogenic vertigo” [6]. This concept is beginning to gain traction among other investigators [2]. Thompson-Harvey and Hain [9] noted that a carefully constructed questionnaire was unable to distinguish CV from migraine-associated vertigo. There are actually two possibilities covered by this idea.

The first possibility is that neck problems may trigger migraine [61,62,63,64,65], and migraine can cause vertigo [66,67,68]; on this hypothesis, neck problems are the initial trigger for migraine, and migraine in turn causes vertigo.

The second possibility is that migraine may manifest with both neck pain [64,69,70,71,72,73,74,75,76] and vertigo [66,67,68]; on this hypothesis, migraine is the common underlying etiology of both symptoms.

The idea of a relationship between migraine, neck pain, and vertigo is attractive in the sense that it suggests a unifying diagnosis, but its limitation is that it exchanges one untestable diagnosis (CV) with another (migraine-associated vertigo). However, one merit of this theory is that it opens a potential avenue for treatment (migraine prophylaxis).

## 6. Attempts at Developing Objective Diagnostic Tests and Their Failures

The range of hypothesized pathophysiological mechanisms for CV has led to a corresponding range of attempts at developing a test for CV. Some of the main ones are summarized in Table 2. We shall review each of these in the following sections.

### 6.1. Imaging

This usually is employed to investigate the possibility of dynamic vascular compromise. As discussed earlier, this may help corroborate clinical suspicion for pathologies such as rotational vertebral artery syndrome, but since normal subjects can exhibit similar imaging, the finding is not specific for RVAS [5,28].

### 6.2. Posturography

Numerous studies have explored computerized dynamic posturography in patients with possible CV [77,78,79,80,81,82,83,84]. However, “increased postural sway is a nonspecific finding that is also evident in patients with vestibular injury” [8]. Moreover, “postural instability can be simulated” [5].

### 6.3. Oculomotor Studies

Since relevant multisensory inputs converge at the vestibular nuclei [1,8,21,47] whence efferent pathways project to the oculomotor nuclei, it is logical to explore whether CV manifests with oculomotor abnormalities. A variety of oculomotor findings have been reported in patients with clinical histories compatible with CV [85], including abnormalities in smooth pursuit, abnormal caloric responses, spontaneous and positional nystagmus [8], latent nystagmus, and abnormalities on rotatory chair testing [86]. Unfortunately, these have proven neither sensitive nor specific for CV. We will review several oculomotor tests that have been explored: smooth pursuit, optokinetic after-nystagmus, and the cervico-ocular reflex.

### 6.4. Smooth Pursuit

Some investigators report abnormalities of smooth pursuit in whiplash patients with dizziness [52,87]. However, “Smooth pursuit is a complex multiple input system that is vulnerable to cognitive variables, age, and sedation. Neck pain and secondary gain, both disruptive of cognition, would also seem highly likely to influence performance of smooth pursuit. For these reasons, due to an intrinsic issue with specificity, it seems unlikely that any smooth-pursuit test could be of general utility for the diagnosis of cervical vertigo” [6].

### 6.5. Optokinetic After-Nystagmus

It has been hypothesized that optokinetic after-nystagmus may be abnormal in CV patients [88]. However, “Optokinetic after-nystagmus is difficult to obtain in humans and is generally of small velocity even in normal subjects. This makes it unlikely that this test could be sufficiently sensitive to be useful in cervical vertigo” [6].

### 6.6. Cervico-Ocular Reflex on the Head-Still Trunk-Rotates Protocol

One oculomotor testing protocol merits special attention. Fixing the head in space (thus neutralizing labyrinthine input) while oscillating the trunk underneath [46,49,50,51] should, in theory, come close to selective manipulation of proprioceptive cervical input, and an output such as eye movements could then be analyzed. A passive version of this test has been studied [89]. An active version of this test—in which the subject has to attempt to keep the head stationary by pointing a “gunsight” laser at a stationary target while the trunk rotates beneath—has also been studied [90].

While this test strikes us as very logical, it has proven neither sensitive nor specific for CV [91]. In greater detail, the “test of cervical rotation, actually a test of the cervico-ocular reflex, consists of rotating the body about the earth’s vertical axis, while keeping the head still in space, and evaluating for nystagmus. This procedure has not been widely accepted… The cervico-ocular reflex also appears in other conditions, such as bilateral vestibular loss thus even if it were sufficiently sensitive, the finding of a cervico-ocular reflex could not be… a specific test for cervical vertigo” [6]. Moreover, Brandt [1] notes that “Cervical nystagmus also occurs in healthy subjects,” citing Norre [92]. It further turns out that even in normal subjects it is possible to induce asymmetry in the vestibulo-ocular reflex by passive sustained turning of the head-on-trunk [93].

A more detailed explanation of this from Wrisley et al. [8] is that:

*“The neck torsion nystagmus test, or head-fixed, body-turned maneuver is considered by some to identify cervicogenic dizziness* [94]. *This test requires the head of the patient to be stabilized while the body is rotated underneath* [92,95]. *Theoretically, the neck proprioceptors are stimulated while the inner ear structures remain at their resting state* [92]. *Nystagmus is elicited in a positive test. However, this test has not been demonstrated to be specific for cervicogenic dizziness. Oosterveld et al.* [96] *reported that 64% of 262 patients with neck pain who presented to an otolaryngology department post-whiplash had nystagmus elicited with the head-fixed, body-turned maneuver. On the other hand, it has been demonstrated that up to 50% of subjects without cervical spine pathology have also demonstrated nystagmus with the head-fixed, body-turned maneuver* [92,94,97]. *A positive response (nystagmus) may not indicate pathology, but may instead be a manifestation of the cervical ocular reflex* [92]*”.*

### 6.7. Why Has Testing for CV Failed?

It seems that it should be possible to isolate individual sensory inputs, with the relevant one for CV being proprioception. Yet, this has proven challenging to study. A few comments from investigators allude to this problem.

*“Postural control is achieved through a multisensory control mechanism involving visual, vestibular and somatosensory information. These inputs are all interconnected allowing compensation of dysfunctions but making it very challenging to study the cues of one particular system without interference of another”* [98].

*“The neck not only modulates body posture, but it also stabilizes the head in space by cervicocollic reflexes, which are similarly integrated with vestibulocollic reflexes. In healthy human beings, neck reflexes form part of the multisensory postural control mechanism, thus making it impossible for the clinician to carry out a selective test of neck function by simple postural maneuvers”* [1].

Essentially, it has proven difficult to manipulate selectively an individual input in complete isolation from the other inputs.

Thus, the failure to develop a test that is specific and sensitive for CV is due, at least in part, to the complex organization of the system, and in particular to its multi-modal nature. The input (perception of movement and of orientation) and output (execution of movement for maintenance of equilibrium) is a process that involves multimodal sensory afferents (vestibular, vision, proprioception), integration of those inputs, and multimodal efferents (oculomotor, somatomotor). This system has advantages and disadvantages. An example of an advantage for a patient is that the multiple inputs are not completely overlapping, but insofar as they do overlap and provide concordant information, this redundancy makes the system more resilient. An example of a disadvantage for a patient is that when the sensory inputs are discordant, the resulting mismatch can be perceived as vertigo. A chief disadvantage for the investigator is that it is difficult to manipulate selectively a single input while maintaining the other inputs constant.

If a successful test were to be developed, what might it look like?

A general principle of sensory input is that biological sensors are better at detecting change (dynamic stimulation) than stasis (constant stimulation) [99]. This general principle underlies Brandt’s idea that “a multisensory mismatch would be expected to result in CV… the resultant mismatch would be maximal during active head movements (when expected and actual reafferent input do not match)” [1], which in turn leads to his suggestion that “Since assessment of all these measures under static conditions has so far proven inconclusive, further investigations should focus on dynamic studies” [1].

Given these considerations, if a test is ever devised that successfully identifies cervicogenic vertigo (and distinguishes it from other diseases), it seems likely that the test will involve dynamic input, probably in the form of some change in position. Until such a test is developed, we need to bear in mind that patients with suspected cervicogenic vertigo usually perceive their symptoms to be more pronounced during movement.

### 6.8. Dynamic Testing May Be More Sensitive Than Static Testing, but…

However, a test that involves a change in position will still run the risk of stimulating multiple inputs. Positional changes can trigger vertigo in diseases other than putative CV, of course. Yacovino and Hain [6] pointed out that four out of the five cases reported in the very paper that gave us the term “cervical vertigo” [7] sound much more like benign paroxysmal positional vertigo (BPPV). Let us review the cases described by Ryan and Cope.

*Case 1: “A man, aged 57, had been in excellent health… While he was building a wall a brick had struck him in the head; his neck was flexed at that time and the blow jarred his neck to one side… On lying down that night he had a sudden and fairly severe attack of vertigo—‘everything started going round and I felt sick.’ He did not vomit, and after a minute or two the vertigo subsided; but the neck pain did not allow him much sleep… He was… placed flat on a couch, and his head and trunk lowered over the end… At once he complained of vertigo, and we noticed that nystagmus appeared in association with this symptom. The vertigo and nystagmus occurred irrespective of whether the head was to the left, central, or to the right, and disappeared if the patient maintained his position for a little time.”*

*Case 2: “A man, aged 60, was involved in an explosion… and he subsequently complained of pain and paresthesiae in the arms… He was subjected to manipulation, and thereafter slow neck traction was applied… Three days later on getting up in the morning he had an acute attack of vertigo and fell to the left. There was no vomiting, sweating, or syncope. On trying to move to the right or the left he experienced vertigo and was unable to walk straight. He stayed in bed for a week and then got up cautiously, finding that the vertigo was brought on only by rapid movement of the head. The symptoms slowly diminished and had disappeared within two weeks.”*

*Case 4: “A married woman, aged 45… fell on to the base of her spine and also injured the back of her head… She… had slight pain in the occipital region. About three weeks after this injury she was wakened in the night by a throbbing sensation in the right side of her neck; and in the morning she sat up in bed, felt her head swimming, and fell to the left. This sensation, which was momentary, was accompanied by slight nausea. About ten minutes later she ‘felt fine,’ and got up to do her housework. Just before luncheon she looked up at the warming-rack and then bent down to attend to the stove, and immediately the kitchen ‘seemed to spin round’ to the left, and she fell on her left side.”*

*Case 5: “A married woman, aged 40, slipped and fell on the back of her head… The next day she had a severe pain in the occipital region… Some time later she had a severe attack of vertigo, with a feeling that her environment was rotating in a clockwise direction. Since then the attacks had occurred twice a week, and on two occasions she had fallen to the ground and bruised herself.”*

These cases all sound like attention was being drawn to the neck due to the circumstances in which the patient was injured, but the descriptions of the vertiginous symptoms themselves and their positional triggers are far more suggestive of benign paroxysmal positional vertigo; the cervical symptoms are more likely to be “distractors.” In other words, these were probably patients with BPPV that happened to be occurring in the context of trauma to the head or neck. Since BPPV is the most common cause of vertigo [100], it is statistically likely that its occurrence will coincide with that of other diseases (such as neck pain, head trauma, etc.).

Bear in mind that when Ryan and Cope were writing in 1955, benign paroxysmal positional vertigo had barely been described. In 1920, Robert Bárány [101] described a case of a young woman who had been suffering from vertiginous episodes triggered by lying on her right side. Numerous similar cases were described subsequently, and in 1952, Margaret Ruth Dix and Charles Skinner Hallpike [102,103] described their own cases of “positional nystagmus,” noting that symptoms (and simultaneous nystagmus) were induced “by a critical position of the head in space.” Ryan and Cope appear to have been aware of the work of Dix and Hallpike since they cite one of their 1952 papers, but in reading Ryan and Cope’s paper with hindsight, it seems likely that the “cervical circumstances” in these cases were distractors from the true pathology.

This reinforces the point made by other investigators that “Benign paroxysmal positional vertigo (BPPV) is often misdiagnosed as cervical vertigo” [2], or more generally, “Lesions of the vestibular organs, particularly the otolithic organs, after whiplash injuries are probably underestimated by attributing dizziness and vertigo symptoms mainly to cervical damage and lesions of the central nervous system” [18]; see also Dispenza et al. [104].

The relevance for this volume on positional vertigo is that in a patient with positional vertigo, before entertaining the evasive diagnosis of CV, be sure to evaluate for common diagnoses, such as BPPV.

## 7. Many Patients Preliminarily Diagnosed with CV Are Found to Have Other Disorders

Mistaking BPPV for CV is a particularly good illustration of why, when a diagnosis of CV is being considered, one must maintain a broad differential.

Yacovino and Hain comment that, “Many patients preliminarily diagnosed with such a disorder are ultimately found to have other pathologies” [6]. Brandt states this more forcefully: “Reliable and well-established signs and tests can support a convincing alternative diagnosis in almost all patients presenting with vertigo” [1].

This serves as a reminder that CV remains a diagnosis of exclusion.

## 8. A Diagnosis of Exclusion

Given the difficulty of devising a “proof positive” test for CV, and the repeatedly cited observation that most cases preliminarily diagnosed with CV are ultimately found to have a different cause, most reviews come to the conclusion that CV is a diagnosis of exclusion [2,3,6,8].

Taking the “diagnosis of exclusion” criterion with the definitions, conditions, and assumptions mentioned earlier brings us back to Wrisley’s description that “the diagnosis of cervicogenic dizziness is suggested by (1) a close temporal relationship between neck discomfort and symptoms of dizziness, including time of onset and occurrence of episodes, (2) previous neck injury or pathology, and (3) elimination of other causes of dizziness” [8].

## 9. What Constitutes an Adequate Workup?

If CV is a diagnosis of exclusion, then the question arises: what else needs to be excluded? Beyond a thorough history and physical examination, there is no consensus on what constitutes an adequate workup to exclude alternative diagnoses.

Practically, since inner ear disturbances are so common, it is reasonable to consider a screening otovestibular workup. At the discretion of the clinician, this could include:

- Ocular vestibular evoked myogenic potentials and videonystagmography.
- If available, rotatory chair testing and computerized dynamic posturography may also be appropriate.
- If the patient’s neck will tolerate it, consider cervical vestibular-evoked myogenic potentials and video head impulse testing.

If neck rotation provokes symptoms other than vertigo, or reveals physical examination signs of brainstem dysfunction, then imaging is probably warranted. At the discretion of the clinician, this could include:

- Vascular imaging, preferably dynamic, such as CTA, MRA, or dedicated catheter angiography. There is some evidence that transcranial Doppler ultrasound may also have some role [105].
- Imaging of the bony structures (usually by CT) and soft tissue structures (usually by MRI) of the cervical spine.

## 10. Treatment

The controversy surrounding CV has not prevented clinicians from attempting to treat it, though the optimal therapeutic protocol is uncertain since the underlying mechanism of the disease remains unclear.

### 10.1. Physical Therapy

There has been extensive reporting on physical therapy for the neck as treatment for CV [78,106,107,108,109,110,111,112,113], though, “Regarding the treatment of proprioceptive cervical vertigo, in which pain and imbalance or vertigo are the limiting symptoms, the quality of published studies in the current literature is poor” [6].

Some authors suggest that a multimodal approach to treatment may be warranted, as evidenced here: “A combined approach is likely to best address the perpetuation of a vicious cycle of events where secondary adaptive changes in the sensorimotor control system could lead to altered cervical muscle function and joint mechanics further altering cervical afferent input… Physical therapy interventions such as pain management, manipulative therapy, active range-of-motion exercises, and exercises to improve neuromuscular control will all be important in reducing possible causes of altered afferent cervical input and subsequent disturbances to sensorimotor control” [3]. It may also be helpful to incorporate treatment from other disciplines, such as vision therapy.

Although vertigo is one of the cardinal symptoms of CV, “Vestibular physical therapy is not a substitute for physical therapy for the neck” [5].

### 10.2. Why Does PT Work at All?

If we do not yet know the pathophysiological mechanism underlying CV, then applying physical therapy in this clinical scenario is treating blindly, and seems unlikely to correct the problem by chance; and yet, the physical therapy literature generally describes encouraging outcomes. How can this be?

Whichever factor initiates the process that manifests with one of the symptoms (vertigo or neck pain) may in turn provoke the other, leading to the vicious cycle of a positive feedback loop. Patients who are dizzy from any cause tend to develop neck stiffness [6,15], and neck problems causing vertigo defines CV. In other words, these phenomena may exacerbate each other; “interconnections between the cervical proprioceptors and the vestibular nuclei may contribute to a cyclic pattern, such that cervical muscle spasms contribute to dizziness and dizziness contributes to muscle spasm” [8].

Whether the neck symptoms are the cause or the consequence of vertigo, any treatments that reduce neck pain and normalize cervical muscle tone and joint mobility—basically any treatments that normalize cervical mechanics [85,114]—will interrupt this positive feedback loop, thereby increasing the opportunity for recovery. This idea is reflected in Brandt’s comment that “If CV exists, appropriate management is the same as that for the cervical pain syndrome” [1].

### 10.3. Medication

Most pharmacologic attempts at management have included muscle relaxants, though there are no good data to suggest that this is effective. On the theory of “migraine-associated cervicogenic vertigo,” a trial of migraine prophylaxis may be reasonable. There has been some exploration of other approaches, such as moxibustion [115] and onabotulinum toxin [116].

### 10.4. Surgery and Other Invasive Procedures

A variety of invasive interventions for CV have been explored, including cervical medial branch blocks [117,118], percutaneous cervical nucleoplasty [119], radiofrequency ablation nucleoplasty [120], percutaneous laser disc decompression [121], intervertebral disc replacement [122], and surgery for cervical spondylosis or disc herniation [98]. Case series of the various invasive treatments for presumed CV generally report variable outcomes. Given the uncertainty in establishing the diagnosis, and the risks of invasive procedures, we would view invasive procedures as a last-resort approach.

### 10.5. Alternative Therapies

Dry needling [123] and acupotomy [124] have been explored, with limited data.

## 11. Summary and Conclusions

Vestibular clinicians often encounter patients with neck symptoms and vertigo in whom it may seem logical to postulate a causal relationship between the two symptoms.

The most popular theory is that cervicogenic vertigo is due to an abnormality in cervical proprioception. Etiologies such as vascular compromise, even if sometimes correct, probably account for only a modest proportion of cases.

There have been numerous attempts at developing a test for cervicogenic vertigo; none appears sufficiently sensitive or specific, and none has gained wide acceptance. It seems likely that this failure is at least in part due to the fact that the system in question involves multimodal sensory integration, and in practice it is difficult to manipulate selectively a single sensory modality while leaving the other modalities unchanged.

In the absence of a confirmatory test for CV, it remains a diagnosis of exclusion. Depending on the clinical scenario and findings on physical examination, additional workup may include otovestibular testing and imaging.

Physical therapy for the neck has been studied more than other treatment modalities, and is usually described as having favorable outcomes. Given our ignorance of the underlying mechanism of disease, it is difficult to understand why physical therapy should be helpful, but one possibility is that such therapy normalizes cervical mechanics and thereby interrupts the maladaptive positive feedback loop in which each symptom (vertigo, neck pain) exacerbates the other.

Cervicogenic vertigo more commonly provokes symptoms during positional changes and other movements. Such movements can also provoke other forms of vertigo, so when formulating a differential diagnosis for CV, it is prudent to keep in mind common causes as well, such as benign paroxysmal positional vertigo.

## Figures and Tables

**Table 1:** A selection of pathophysiological mechanisms proposed for CV.

Mechanism	Comments
Hypoperfusion	Neck movements may result in physical compromise of arteries in the neck by torque or compression, resulting in reduced blood flow to the brainstem. Another possibility is that neck movements may induce autonomic responses (e.g., through stimulation of carotid baroreceptors), also resulting in hypoperfusion.
Anatomic	In an individual with craniocervical instability, neck movements may provoke brainstem compression.
Oculomotor	Neck movements, through a variety of mechanisms, may induce abnormal oculomotor responses.
Proprioception	In a diseased neck, neck movements may generate aberrant proprioceptive signals.
Motoric	In a diseased neck, neck movements may incorrectly modulate efferent motor signals.
Migraine	Neck disease may trigger migraine, and migraine can cause vertigo. Another possibility is that migraine causes both neck discomfort and vertigo.

**Table 2:** A selection of tests intended to help diagnose CV.

Test	Comments
Imaging	Imaging is usually intended to detect vascular compromise triggered by head-on-neck rotation, and thus include various types of angiography (CTA, MRA, transcranial Doppler ultrasound, dedicated catheter angiography).
Posturography	Computerized dynamic posturography was originally designed to assess vestibular, visual, and proprioceptive contributions to an individual’s unsteadiness. Its utility in CV has also been explored.
Oculomotor studies	A range of oculomotor findings have been reported in CV, including abnormalities in spontaneous nystagmus, smooth pursuit, caloric responses, optokinetic after-nystagmus, and cervico-ocular reflex responses.
